# Mechanism of Qingchang compound against coccidiosis based on network pharmacology-molecular docking

**DOI:** 10.3389/fvets.2024.1361552

**Published:** 2024-03-01

**Authors:** Zhiqiang Yan, Chunlin Chen, Shaoqin Zhai, Hongmei Tang, Maixun Zhu, Yuandi Yu, Hua Zheng

**Affiliations:** ^1^Chongqing Academy of Animal Sciences, Rongchang, China; ^2^Chongqing Research Center of Veterinary Biological Products Engineering Technology, Chongqing, China

**Keywords:** Qingchang compound, network pharmacology, molecular docking, experimental verification, main components, core target

## Abstract

The aim of this study was to investigate the anti-*Eimeria tenella* mechanism of Qingchang Compound (QCC) and provide a basis for its clinical application. The active ingredients, active ingredient-disease intersection targets, and possible pathways of QCC for the treatment of chicken coccidiosis were analyzed, the binding ability of pharmacodynamic components and target proteins was determined by network pharmacology and the molecular docking, and a model of infection with coccidiosis was constructed to verify and analyze the mechanism of action of QCC against coccidiosis. Among the 57 components that met the screening conditions, the main bioactive components were quercetin, dichroine, and artemisinin, with IL-1β, IL-6, IL-10, IFN-γ, and IL-8 as the core targets. Simultaneously, the KEGG signaling pathway of QCC anti-coccidiosis in chickens was enriched, including cytokine-cytokine receptor interactions. The results showed that the main pharmacodynamic components of QCC and the core targets could bind well; artemisinin and alpine possessed the largest negative binding energies and presented the most stable binding states. In addition, *in vivo* studies showed that QCC reduced blood stool in chickens with coccidiosis, restored cecal injury, and significantly reduced the mRNA and protein expression levels of IL-1β, IL-10, and IFN-γ in ceca (*p* < 0.01). Our results suggest that the main active ingredients of QCC are artemisinin and alpine and its mechanism of action against coccidiosis may be related to the reduction of the inflammatory response by acting on specific cytokines.

## 1 Introduction

Chicken coccidiosis is a protozoan disease caused by coccidian parasites belonging to the phylum Apicomplexa, class Sporozoa, order Eucoccidiorida, family *Eimeriidae*, and genus *Eimeria*. The main causative agents of chicken coccidiosis are seven species of *Eimeria*, namely *Eimeria tenella*, *E. necatrix*, *E. brunetti*, *E. maxima*, *E. acervulina*, *E. mitis*, and *E. praecox*, among which *E. tenella* is the most pathogenic. This disease primarily affects young and juvenile chickens (1–4). It has been reported that the annual economic loss to China’s chicken industry due to coccidiosis is up to 5 billion yuan, and drug expenditure alone is approximately 1.56–1.95 billion yuan (5).

Although, the chemotherapeutic drugs (Diclazuril and Sulfonamides) were mainly agents, it had a severe resistance in coccidia isolated from chicken farms, manifesting as broad, crossover, or multidrug resistance (6). Therefore, researchers have conducted in multiple directions, including live vaccines, recombinant vaccines, and medicinal plants (7–9). However, live vaccines have problems of poor stability, easy virulence regression, and uneven immunity, thus the application of live vaccines for coccidia needs further improvement. At present, the development and clinical application of recombinant proteins targeting coccidia require a long time. Traditional Chinese medicine is widely used in veterinary clinics because it is difficult for microorganisms to develop drug resistance to these widely sourced herbal prescriptions. Hence, the research and development of veterinary traditional Chinese medicine anti-coccidial drugs aligns with the market demand and national policy.

The main characteristic of veterinary Chinese medicinal compounds is the combination of multiple components and targets; however, traditional research of compound therapeutics has mainly involved a single target and pathway. Research on the mechanisms of traditional Chinese medicines against parasite has mainly focused on the direct effects of drugs on the activation of the host’s immune function. The direct drug effects include recognition, movement, adhesion, invasion, and intracellular development, whereas the activation of the host’s immune function includes both specific and non-specific immunity, making it difficult to fully explain the pharmacological effects and molecular mechanisms of Chinese veterinary drug compounds. In recent years, network pharmacology has been widely used to reveal the pharmacodynamic components, action of signaling channels, and compatibility connotations of Chinese veterinary drugs with the overall research mode of “multi-component-multi-target disease” (10–12). Currently, research on the anti-coccidiosis effect of QCC in chickens is mostly limited to pharmacodynamic studies, and there are few reports on the anti-coccidiosis mechanism(s) of action of this herb. The traditional methods for studying the possible mechanism of action of veterinary Chinese medicine against coccidiosis are tedious, time consuming, and expensive. In contrast, the use of network pharmacology and molecular docking in this regard presents significant advantages (efficient, accurate, and comprehensive) (13). In this study, the main pharmacodynamic components, core targets, and enriched signaling pathways of QCC were analyzed using modern Chinese medicine network pharmacology and molecular docking, the core targets of the drug were verified through animal studies, and the anti-coccidiosis mechanism of action of QCC was determined.

## 2 Materials and methods

### 2.1 Network pharmacology analysis of the anti-coccidiosis effect of QCC in chickens

#### 2.1.1 Construction of a drug-active ingredient-target network for QCC

The filtering conditions for the TCMSP database were Oral Bioavailability (OB) ≥ 30% and Drug-likeness (DL) ≥ 0.18. Through literature review, we identified the active components of four Chinese herbs—Artemisiae annuae herba, Dichroae radix, Agrimonia pilosa, and Sanguisorbae radix—and supplemented or cross-verified the data retrieved. Target information for these active components was downloaded from the TCMSP database. Component target proteins and gene names were matched using UniProt.^1^ The drugs, active components, and potential targets were imported into Cytoscape 3.8.2 software (Bloomage BioTechnology Corporation Limited, China) to construct a drug-active component-target network to identify important candidate components.

#### 2.1.2 Screening of targets for the prevention and treatment of chicken coccidiosis with QCC and construction of a protein–protein interaction network

In the GeneCards,^2^ OMIM,^3^ and NCBI^4^ databases, we conducted keyword searches using the terms “Avian Coccidiosis,” “Coccidiosis in Chicken,” and “Chicken coccidia” to obtain targets related to coccidiosis in chickens. Species filtering was then performed using UniProt, and a Venn diagram was generated online.^5^ The targets of Artemisiae annuae herba, Dichroae radix, Agrimonia pilosa, Sanguisorbae radix, and chicken coccidiosis were imported separately. The intersection of these sets was used to identify common targets, which serve as targets for treating chicken coccidiosis with QCC. The intersecting targets obtained from the Venn diagram were imported into the String database.^6^ The species “Gallus” was selected, and a minimum interaction score of “medium confidence (0.400)” was chosen. After the protein–protein interaction (PPI) network was obtained, a TSV-format file was downloaded. This file was imported into Cytoscape 3.8.2 to construct a PPI network. The “Analyze Network” function was used to perform network analysis, calculating the degree values for all nodes in the network. Targets that ranked the highest based on their degree values were selected and used for subsequent molecular docking verification.

#### 2.1.3 Gene ontology enrichment analysis and KEGG pathway enrichment analysis for targets of QCC in chicken coccidiosis

Gene Ontology (GO) and Kyoto Encyclopedia of Genes and Genomes (KEGG) pathway enrichment analyses were performed on the potential target genes through the DAVID website.^7^ The input for potential targets was restricted to chicken species. The resulting GO analysis was filtered based on a *p*-value of less than 0.05, and the KEGG analysis was filtered based on a *p*-value of less than 0.01. The results were sorted according to the number of enriched genes, and the top 20 entries were selected for both. Bubble charts and bar graphs were created to identify the main biological processes (BP), primary cellular components (CC), and principal molecular functions (MF) involved, as well as key signaling pathways implicated.

#### 2.1.4 Molecular docking verification of core active components and targets of QCC

The 3D structures of the components of Artemisiae annuae herba, Dichroae radix, Agrimonia pilosa, and Sanguisorbae radix were obtained from the PubChem database and converted to the moL2 format using OpenBabel. They were then imported into PyMOL for addition of hydrogen, charging, and other treatments. The protein conformations were screened using the RCSB PDB database.^8^ The selection criteria included having a well-defined protein structure obtained through X-ray crystallography with a crystal resolution smaller than 3A. These proteins were then processed to remove water and small molecules and treated with hydrogen addition, charge addition, and non-polar hydrogen merging in Autodock Tools 1.5.6. Docking calculations between large and small molecules were performed using AutoDock Tools. Receptor–ligand pairs were sorted and screened based on binding free energy (affinity in kcal/mol). A compound is considered to spontaneously bind and interact with a protein if its binding energy is <0 kcal/mol, with lower-energy conformations being more stable. Generally, a binding energy of <−1.2 kcal/mol indicates good binding efficiency (14). Visualization was performed using PyMOL 2.3.2 software.

### 2.2 Experimental validation of QCC against chicken coccidiosis

#### 2.2.1 Preparation of QCC and tender *Eimeria* oocysts

A precise amount of QCC (Artemisiae annuae herba, Dichroae radix, Agrimonia pilosa, and Radix Sanguisorbae = 5:2:2:1) was weighed and soaked in distilled water for 0.5 h, followed by extraction in 75% ethanol for 1 h, and subsequent water extraction for 1 h. The extracts from both processes were combined and concentrated to yield a solution with a concentration of 1.0 g/mL of raw medicine, which was then set aside for later use.

Tender *E.* oocysts were obtained from the Chongqing Academy of Animal Science. Gavage feeding of sporulated oocysts was performed in chickens to revitalize the oocysts. Fecal samples from the chickens were collected upon the appearance of bloody feces to isolate the oocysts. Harvested *E.* oocysts were cultured in 2.5% potassium dichromate solution at 28°C for 3 days (10). During this period, oocyst status was monitored. Culturing was halted when sporulation and hatching rates exceeded 90%, and the sporulated oocysts were collected and set aside.

#### 2.2.2 Experimental grouping and treatment

One-day-old Ross broiler chicks purchased from a poultry farm in Chongqing were allowed *ad libitum* access to food and water. The feed did not contain any additives. After reaching 16 days of age, the chicks were transferred to flame-sterilized cages.

Thirty 16 days-old Ross broiler chicks were randomly divided into three groups: Control, Model, and QCC. Except for the Control group, the other two groups were orally gavaged with 1 × 10^4^ sporulated oocysts per chicken. Five days post inoculation, the QCC group was orally administered QCC extract at a dose of 2.4 g/kg. An equivalent volume of physiological saline was administered to the Control and Model groups. The treatment was administered once daily for a continuous period of 7 days. All chickens had *ad libitum* access to food and water, and the feed provided did not contain any additives.

#### 2.2.3 Effect of QCC on clinical manifestations and cecal tissue of chickens with coccidiosis

During this study, the clinical manifestations of the chickens were observed and recorded, including their mental state, feeding, fur, movement, and fecal state. At the end of the study, all chickens were dissected, and the ceca was collected, fixed with 4% paraformaldehyde, embedded in paraffin, sliced, and stained with hematoxylin and eosin. Pathological changes in the tissues were observed under a microscope, and a microscopic imaging system was used to capture images.

#### 2.2.4 Quantification of target gene mRNA levels using qPCR

At the end of the experiment, eight chickens from each group were randomly euthanized. The cecal tissue samples were collected aseptically and immediately stored in liquid nitrogen until further use. Total RNA was extracted from the cecal tissue following the protocol provided in the TRIzol reagent kit. Subsequently, cDNA was synthesized by reverse transcription and used as a template for real-time quantitative PCR (qPCR) analysis. The qPCR products were visualized and photographed using a gel imaging system. The density of the target bands was quantified using image analysis software, and the relative mRNA expression levels of the target genes were calculated using the 2^−ΔΔCt^ method. The primers were designed and synthesized according to gallus IL-1β (NM_204524.1), IL-6 (NM_204628.1), IL-10 (NM_001004414), IL-8 (DQ393272), and IFN-γ (NM_205149.1) gene sequences published in GenBank, all of which were synthesized by Bioengineering (Shanghai) Co., Ltd. The details of the primers used for real-time qPCR amplification are presented in Table 1.

**Table 1:** Primer sequence of qPCR.

Gene	Primer name	Primer sequence	Length of amplification product (bp)
IL-1β	F	GGCCTGAGTCATGCATCGTT	125
	R	ATAAATACCTCCACCCCGACAA	
IL-6	F	GAGGGCCGTTCGCTATTTG	93
	R	GCCATGTGGCAGATTGGTAA	
IL-10	F	ACCTTTGGCTGCCAGTCTGT	167
	R	CAGGTGAAGAAGCGGTGACA	
IL-8	F	CCTGGTTTCAGCTGCTCTGT	129
	R	GGCGTCAGCTTCACATCTTG	
IFN-γ	F	AACCTTCCTGATGGCGTGAA	137
	R	AAACTCGGAGGATCCACCAG	
β-Actin	F	ATGGCTCCGGTATGTGCAA	142
	R	TGGGCTTCATCACCAACGTA	

#### 2.2.5 Statistical analysis

All data were analyzed with SPSS 25.0 statistical software and are presented as means ± SD. One-way analysis of variance (ANOVA) was used to analyze data between groups, and the LSD method was used for pairwise comparisons. Statistical significance was set at *p* < 0.05 and 0.01. The letters a, b, and c indicate *p* < 0.05, and A, B, and C indicate *p* < 0.01.

## 3 Results

### 3.1 Screening results of active components and target sites of QCC

After screening through the TCMSP database, 57 compounds were identified that met the criteria of OB ≥30% and DL ≥0.18. Among them, 22 were from Artemisiae annuae herba, 17 were from Dichroae radix, and an additional compound, dichroine, was included because of its importance, resulting in 18 compounds from Dichroae radix. Additionally, five compounds were identified from Agrimonia pilosa and 13 from Sanguisorbae radix. After deduplication, a total of 51 unique compounds were identified. *Artemisia annua*’s active components corresponded to 210 target sites; Dichroae radix’s active components corresponded to 52 target sites; Agrimonia pilosa’s active components corresponded to 178 target sites; and Sanguisorbae radix’s active components corresponded to 182 target sites. After eliminating the duplicates, 228 unique target sites were identified.

### 3.2 Screening of QCC bioactive ingredients and therapeutic targets for chicken coccidiosis

**Figure 1:**
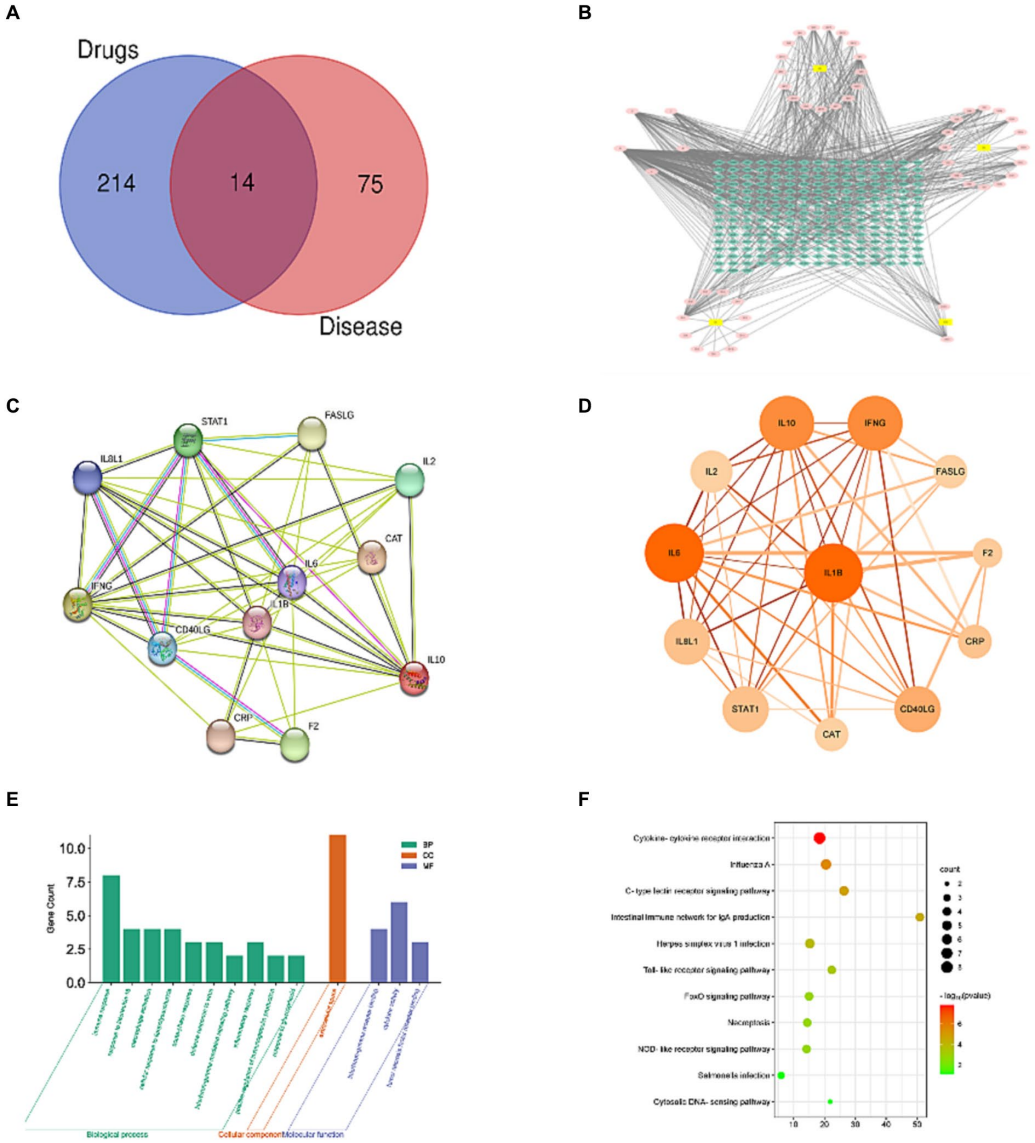
Network pharmacology analysis. **(A)** Venn diagram of intersection of QCC and anti-coccidiosis targets; **(B)** active ingredients-target network of QCC; **(C)** protein–protein interaction (PPI) network within the common targets; **(D)** enrichment analysis of GO biological processes; **(E)** enrichment analysis of pathway signaling.

Following searches in the GeneCards, OMIM, and NCBI databases, 101 disease-target sites were identified. After species-specific filtering, 89 unique disease target sites were identified. Using Venn diagram analyses, 14 intersecting target sites were identified among the active constituents of Artemisiae annuae herba, Dichroae radix, Agrimonia pilosa, and Sanguisorbae radix and disease-specific targets for chicken coccidiosis (Figure 1A). A pharmacological network of drug-active component targets was constructed for Artemisiae annuae herba, Dichroae radix, Agrimonia pilosa, and Sanguisorbae radix. In this network, blue nodes represent potential target sites, pink nodes represent effective compounds, and yellow nodes represent drugs. The graph incorporates 51 compound nodes and 228 potential target nodes. Based on their prominence in the network, the top five compounds were quercetin, kaempferol, luteolin, artemisinin, and berberine. From the perspective of individual compound-target networks and based on the theory of network pharmacology, these five compounds could potentially account for the majority of the therapeutic effects of QCC and may represent the primary active substances in Artemisiae annuae herba, Dichroae radix, Agrimonia pilosa, and Sanguisorbae radix (Figure 1B). An analysis was conducted on the intersecting target sites obtained from both the constituent and disease-specific targets using Venn diagrams on the STRING website, resulting in a PPI network (Figure 1C). The network comprises 12 nodes and 46 edges. The size of each node symbolizes its degree value, whereas the color gradient from light to dark represents betweenness centrality. The thickness and color of the edges indicate the edge betweenness and combined score, respectively. Topological analysis of the PPI network identified hub proteins within this network. Based on their degree values, the top five proteins were IL-1β, IL-6, IL-10, IFN-γ, and IL-8. GO enrichment analysis was performed on potential target genes using DAVID. A total of 31 GO terms were identified, including 27 related to BP, one related to CC, and three related to MF (Figure 1D). The top 10 terms in each category are shown in Figure 1E. KEGG pathway analysis yielded a total of 11 enriched pathways.

### 3.3 Molecular docking validation of core active components and target interactions of QCC

**Figure 2:**
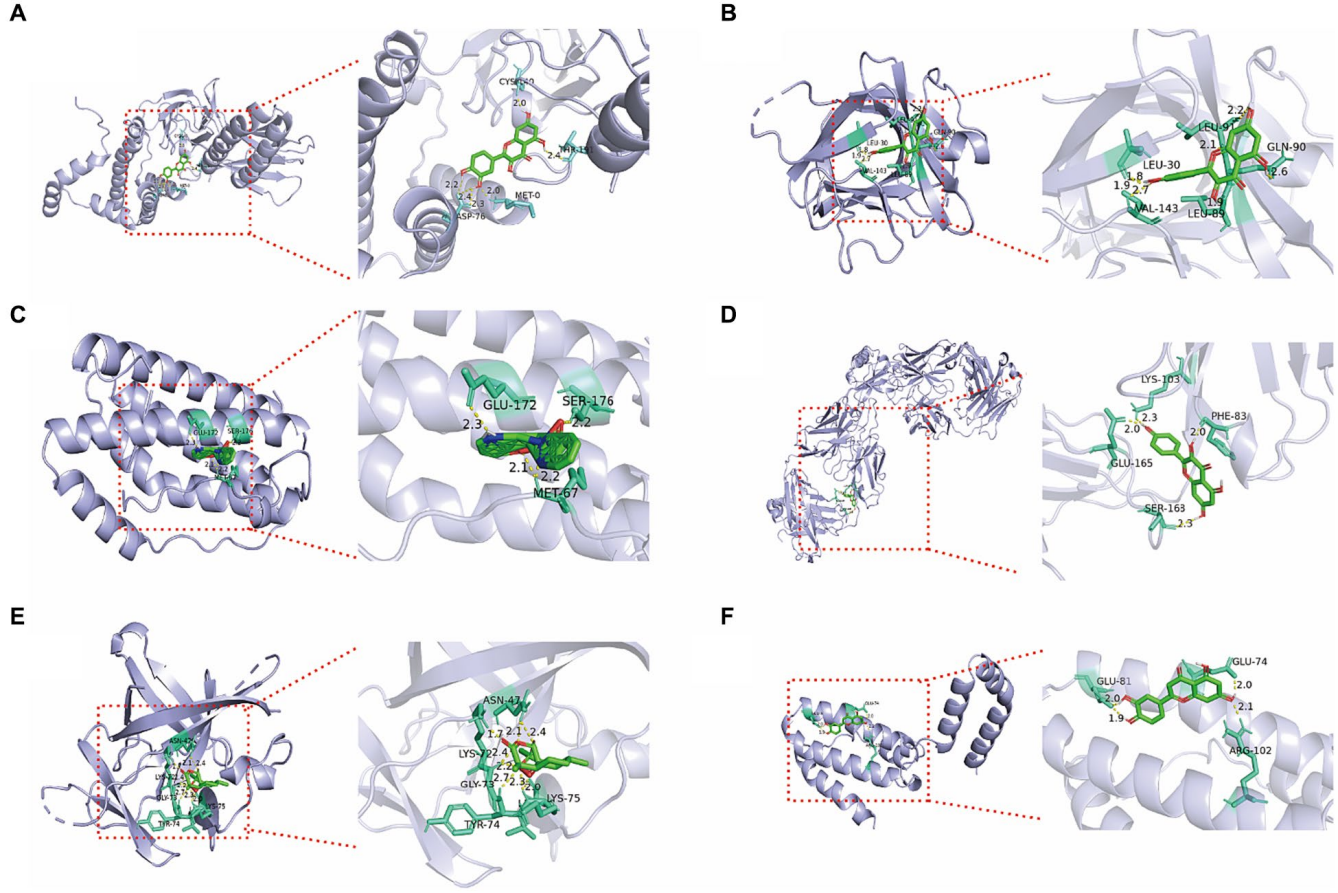
Molecular docking 3D diagram of core active ingredients in QCC with core anti-coccidiosis targets. **(A)** Quercetin-IFN-γ; **(B)** Quercetin-IL-1β; **(C)** Dichroine-IL-6; **(D)** Kaempferol IL-8; **(E)** Artemetin-IL1β; **(F)** Luteolin-IL-10.

Significant compounds such as quercetin, kaempferol, luteolin, artemisinin, and berberine were docked with the core target proteins IL-1β, IL-6, IL-10, IFN-γ, and IL-8. The docking results are listed in Table 2. All docking binding energies were less than −1.2 kcal/mol, indicating that the active components artemisinin, berberine, quercetin, kaempferol, and luteolin from Artemisiae annuae herba, Dichroae radix, Agrimonia pilosa, and Sanguisorbae radix could spontaneously bind with core targets (Table 3). Docking results were visualized using PyMOL software, and detailed docking interactions were observed under magnification (Figure 2). These findings suggested that QCC could exert its anti-coccidial effects through components like artemisinin, berberine, quercetin, kaempferol, and luteolin targeting IL-1β, IL-6, IL-10, IFN-γ, and IL-8.

**Table 2:** Detailed information of ingredients among four herbal medicines in QCC.

ID number	Molecule name	Source
MOL000098	Quercetin	Artemisiae annuae herba, Agrimonia pilosa, Radix Sanguisorbae
MOL000422	Kaempferol	Artemisiae annuae herba, Agrimonia pilosa, Radix Sanguisorbae
MOL000006	Luteolin	Artemisiae annuae herba, Agrimonia Pilosa, Radix Sanguisorbae
MOL000358	beta-sitosterol	Radix Sanguisorbae
MOL000354	Isorhamnetin	Artemisiae annuae herba
MOL000449	Stigmasterol	Dichroae radix, Artemisiae annuae herba
MOL008199	α-Dichroine	Dichroae Radix
MOL008182	Alpha-berbine	Dichroae radix
MOL008193	4,8-dimethoxy-1-methyl-3-(3-methyl-2-oxobutyl)quinolin-2-one	Dichroae radix
MOL005229	Artemetin	Artemisiae annuae herba
MOL001002	Ellagic acid	Agrimonia pilosa
MOL008177	7-4,8-dimethoxy-1-methyl-3-(3-methyl-2-oxobut-3-enyl) quinolin-2-one	Dichroae radix
MOL004609	Areapillin	Artemisiae annuae herba
MOL008178	3-(2-keto-3-methyl-but-3-enyl)-4,8-dimethoxy-carbostyril	Dichroae radix
MOL002235	EUPATIN	Artemisiae annuae herba
MOL007423	6,8-di-c-glucosylapigenin	Artemisiae annuae herba
MOL004083	Tamarixetin	Artemisiae annuae herba

**Table 3:** Binding energy of core-active ingredients in QCC with core anti-coccidiosis targets.

Molecule name	Binding protein	Binding energy (kcal/mol)
Quercetin	IL-1β	−7.22
Quercetin	IFN-γ	−4.7
Quercetin	IL-6	−5.5
Quercetin	IL-8	−3.79
Quercetin	IL-10	−3.78
Kaempferol	IL-1β	−6.79
Kaempferol	IFN-γ	−4.99
Kaempferol	IL-6	−5.58
Kaempferol	IL-8	−4.32
Kaempferol	IL-10	−4.02
Luteolin	IL-1β	−7.11
Luteolin	IFN-γ	−4.94
Luteolin	IL-6	−5.93
Luteolin	IL-8	−4.44
Luteolin	IL-10	−4.68
Artemetin	IL-1β	−8.19
Artemetin	IFN-γ	−6.88
Artemetin	IL-6	−6.85
Artemetin	IL-8	−6.26
Artemetin	IL-10	−6.23
Dichroine	IL-1β	−8.74
Dichroine	IFN-γ	−6.8
Dichroine	IL-6	−8.76
Dichroine	IL-8	−6.01
Dichroine	IL-10	−6.98

### 3.4 Effects of QCC on the clinical manifestations of chickens with coccidiosis

**Figure 3:**
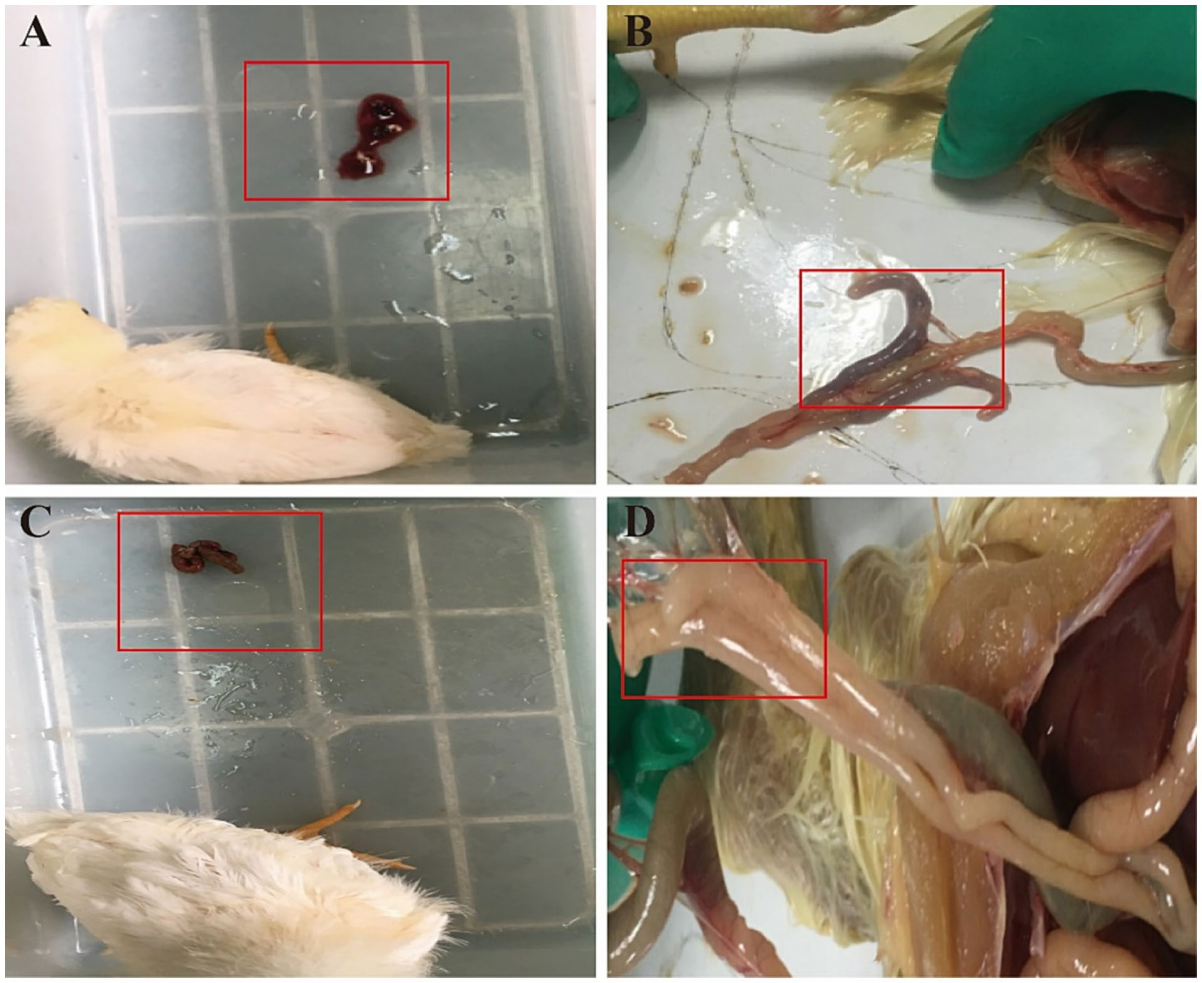
Effect of QCC on clinical manifestations of chickens with coccidiosis. **(A)** The red box indicates that there is a large amount of blood in the feces. **(B)** The red box indicates that the cecum is dark red with clots. **(C)** The red box indicates that a small amount of blood could be found in feces. **(D)** The red box indicates that the cecum is smooth, no swelling, with a smooth outer wall and no obvious blood was found.

Apart from the control group, chickens in both the model and drug treatment groups began to display symptoms of coccidiosis on the 4th day post-inoculation with sporulated oocysts. Symptoms included bloody feces, poor mental state, dull feather coat, decreased food intake, and blood in the cecal cavity. After administering QCC, clinical symptoms improved. The feces became more formed, and only trace amounts of blood were present (Figure 3). The mental state of the chickens improved, food intake returned to normal, and blood in the cecal cavity was not evident.

### 3.5 Effects of QCC on cecal tissue in chickens with coccidiosis

**Figure 4:**
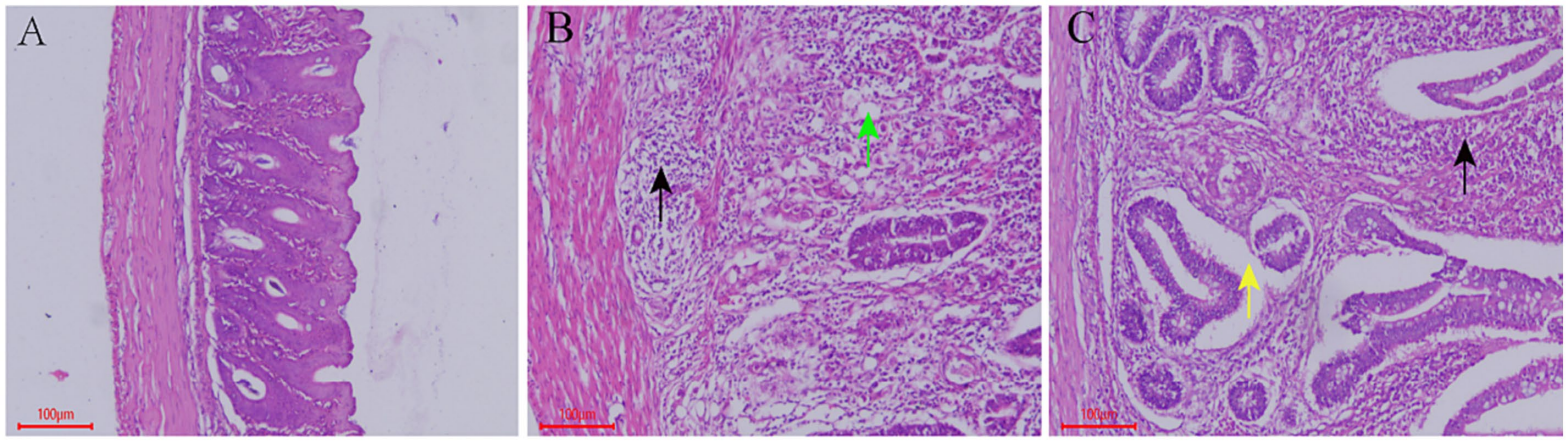
Effect of QCC on Cecal Tissue of Coccidiosis Chickens. **(A)** Control group, HE, Bar = 100 μm, 100×; **(B)** model group, HE, Bar = 100 μm, 100×. A large number of inflammatory cells infiltrate (black arrow), and numerous parasite bodies are seen in glandular epithelial cells (green arrow); **(C)** QCC group, HE, Bar = 100 μm, 100×. Inflammatory cell infiltration (black arrow), glandular epithelial cells detached from the basal portion (yellow arrow).

The cecal tissue structure of chickens in the control group was normal, the cells were neatly arranged and there was no obvious histopathological damage. Cecal tissue from the model group showed a large range of inflammatory cell infiltration, and coccidia bodies were observed in epithelial cells. Further, the mucosal layer was severely damaged. In the QCC group, no coccidiosis was found in the cecum epithelial cells; however, a small area of inflammatory cell infiltration and epithelial cell shedding was observed (Figure 4).

### 3.6 Effect of QCC on the mRNA expression levels of cecal IL-1β, IL-6, IL-8, IL-10, and IFN-γ in chickens with coccidiosis

**Figure 5:**
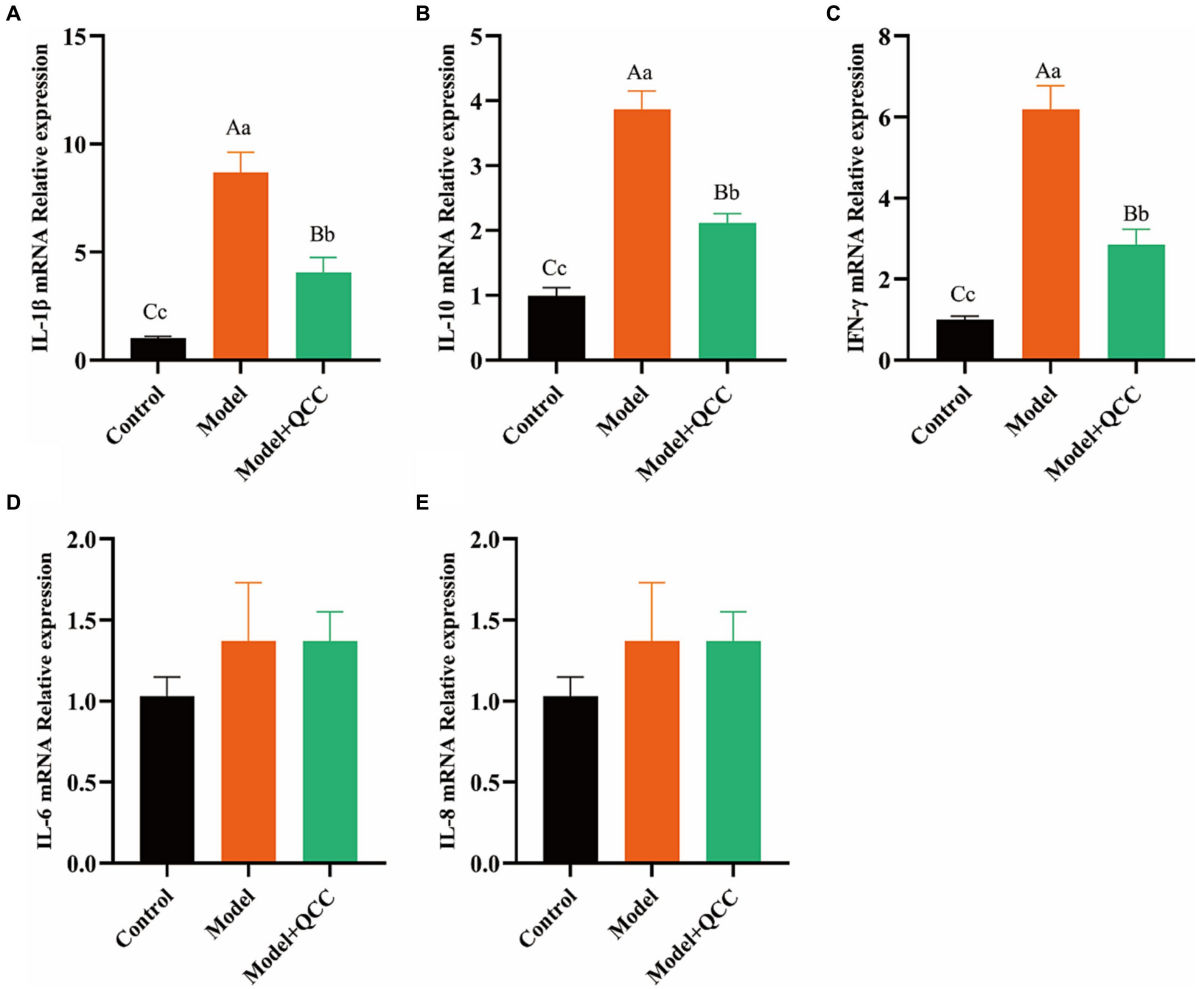
The mRNA expression of coccidiosis after QCC treated in caecum. The mRNA expresses of IL-1β **(A)**, IL-10 **(B)**, IFN-γ **(C)**, IL-6 **(D)**, IL-8 **(E)**. Different lower-case letters on the column mark indicate significant difference (*p* < 0.05), and different capital letters on the superscript indicate extremely significant difference (*p* < 0.01).

At the end of the study, there was no significant difference in the mRNA expression of IL-6 and IL-8 (*p* > 0.05) in cecum tissues. The mRNA expression levels of IL-1β, IL-10, and IFN-γ in the model group were high, and the difference was significant compared with that in the control group (*p* < 0.01). The mRNA expression levels of IL-1β, IL-10, and IFN-γ in the QCC group were reduced, and the difference was significant compared with that in the model group (*p* < 0.01) (Figure 5).

### 3.7 Effect of QCC on protein expression levels of cecal IL-1β, IL-6, IL-8, IL-10, and IFN-γ in chickens with coccidiosis

**Figure 6:**
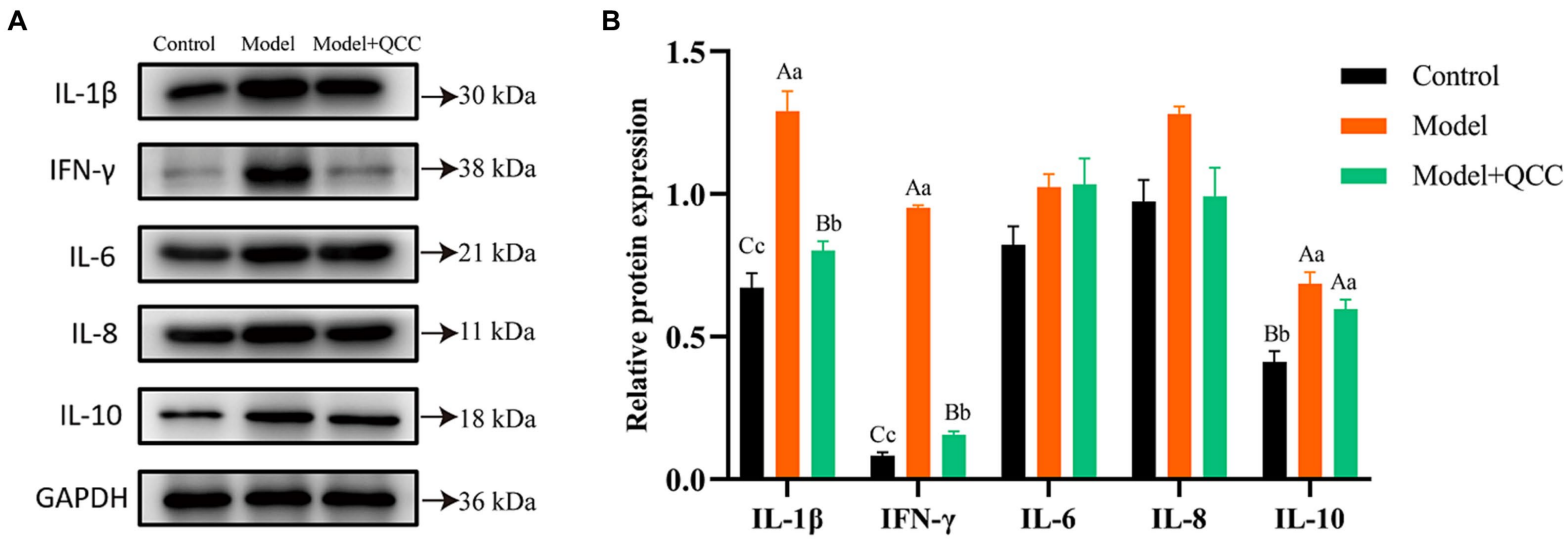
The protein expression of coccidiosis after QCC treated in caecum. The western bolt bar **(A)** and protein expression **(B)** of IL-1β, IL-10, IFN-γ, IL-6, and IL-8. Different lower-case letters on the column mark indicate significant difference (*p* < 0.05), and different capital letters on the superscript indicate extremely significant difference (*p* < 0.01).

At the end of the study, there was no significant difference in the protein expression of IL-6 and IL-8 in the cecum tissues from all the groups (*p* > 0.05). Protein expression of IL-1β and IFN-γ in the QCC group was significantly lower than in the model group (*p* < 0.01). Compared to the control group, the protein expression of IL-10 in the cecum of the model and drug groups increased significantly (*p* < 0.01), but there was no significant difference between the model and QCC groups (*p* > 0.05) (Figure 6).

## 4 Analysis and discussion

Chicken coccidiosis is an acute inflammatory intestinal protozoal disease with high incidence rate and mortality, which is globally prevalent and non-seasonal (15, 16). The use of anticoccidial chemicals, coccidiocides, coccidiostats, and ionophores has been a common practice in modern poultry production for controlling avian coccidiosis. However, the emergence of drug resistance and increasing consumer demand for meat free of residues has prompted the development of alternative control methods (17). In recent years, research has found that some traditional Chinese veterinary medicine, plant extracts, essential oils and probiotics, have significant anti coccidiosis activity with minimal side effects, and low susceptibility to drug resistance, such as Artemisia brevifolia Extract could enhancing the immunomodulatory potential against coccidiosis (18), herbal formula extract highly reduced the coccidian multiplication rate and reduced the severity of intestinal lesions (19). *Citrus sinensis* Essential Oil and Star Anise (*Illicium verum*) Essential Oil supplementation lowered oocysts shedding in faeces and faecal score (20, 21). Among these alternative insecticides, veterinary traditional Chinese medicine has a relatively complete medication theory and history. According to historical experience, some TCMs with “cold taste and bitter” properties, such as Artemisiae annuae herba and Dichroae radix, have antiparasitic effects. Qingchang Compound (QCC) is a traditional Chinese medicine for veterinary use with efficacy against chicken coccidiosis, which has undergone early stage clinical trials alongside Artemisiae annuae herba, Dichroae radix, Agrimonia pilosa, and Sanguisorbae radix (22). Our study preliminarily predicted and validated the activity of the top five active ingredients (quercetin, kaempferol, luteolin, artemisinin, and dichroine) in QCC and their affinity with five core targets (IL-1β, IL-6, IL-10, IFN-γ, and IL-8). Further study revealed that QCC could improve the clinical symptoms of cecal coccidiosis in chickens, reduce cecal tissue injuries, and downregulate the expression of IL-1β, IL-10, and INF-γ.

In this study, the main components of QCC with anti-coccidiosis efficacy in chickens were analyzed by network pharmacology, and the main bioactive components (artemisinin, dichroine, quercetin, kaempferol, and luteolin) exhibit strong anti-parasitic activity. Previous studies have confirmed that the *Artemisia annua* and Atractylodes lancea has significant anti-coccidial effects (23). Artemisinin, the main active component of the traditional Chinese medicine *Artemisia annua*, can induce apoptosis of second-generation merozoites by reducing mitochondrial membrane potential, leading to a remarkable suppression of the upregulated mRNA levels of NF-κB and interleukin-17A in the ceca during *Eimeria* tenella infection (23, 24). In addition, artemisinin can bind to membrane proteins (ATP transporters) in the asexual reproductive stage of plasmodium falciparum to reduce the infectivity of plasmodium malaria parasites (25, 26), and then promote the functional recovery by regulating the body’s inflammatory response. The natural product dichroine and its synthetic derivatives has been proved that can disrupt the activity of synthetase by regulating the gene encoding gluprolyl-tRNA synthetase in plasmodium falciparum (27). The anti-parasitic effects of quercetin, kaempferol, and luteolin are primarily manifested in their ability to inhibit the inflammatory response induced by parasites (28–30). Therefore, we speculate that the anti-coccidiosis effect of QCC is mainly related to artemisinin, dichroine, quercetin, kaempferol, and luteolin, which may be related to their direct inhibition of parasite reproduction and reduction of excessive inflammatory responses.

Molecular docking is an important method for studying the interactions between ligands and receptors, predicting their binding modes and affinities, and searching for candidate compounds based on the docking results (31). We determined that the core targets (IL-1β, IL-6, IL-10, IFN-γ, and IL-8) of QCC can effectively bind with the core pharmacological components, among which artemisinin and alpine showed the largest negative binding energies and most stable binding abilities. And those of IL-1β and IFN-γ to each major component were low, indicating that it could bind spontaneously with the main component. When infected with coccidia, T lymphocytes can secrete various cytokines (the main is IFN-γ, IL-1β), which in turn play a role in anti-infection and immune protection (32). IFN-γ plays a crucial role in resisting intracellular parasitic protozoa, and directly kills parasites or inhibit their growth in the host (33). IL-1β is an important pro-inflammatory factor in the body, which can enhance the host’s resistance to coccidian infection, but can also exacerbate intestinal damage and dysfunction (34). Therefore, it is speculated that the therapeutic substances of QCC can regulate the synthesis and secretion of IL-1β, IFN-γ, and IL-8 through the aforementioned interactions, thereby modulating intestinal inflammation and achieving therapeutic effects against nematode infections. In addition, KEGG analysis showed that these targets are mainly focused on the cytokine receptor interaction and influenza a pathway, indicating that future research should focus on these pathways.

*In vivo* study, IL-1β, INF-γ, and IL-10 mRNA were significantly increased in chicken ceca after infection with coccidiosis, the results were consistent with those of previous studies (35, 36). This paper found that QCC can reduce the expression levels of IL-1β, INF-γ, and IL-10. IL-1β is one of the important pro-inflammatory factors involved in intestinal inflammation, which promotes the production of inflammatory transmitters, the multiplication and differentiation of B cells, and stimulates the expression of immune molecules in body cells, thereby improving the level of humoral and cellular immunity (37). INF-γ is an important active substance that regulates the balance of the Th1/Th2 immune responses and plays an important role in immune regulation (38), which has been reported to be the most important cytokine in the fight against coccidiosis infection (39). IL-10 reduces the degree of intestinal inflammation by inhibiting the release of IL-1, IL-6, and IL-8 (40). Combine with our results, we believe that after treatment with QCC, the body is in a state of low inflammatory response and a low degree of infection, which may be related to the decrease in bloody stool and alleviation of intestinal tissue damage. In the next study, we will verify the effects of the effective ingredients of QCC on the body and investigate whether there is synergy among artemisinin, dichroine, quercetin, kaempferol, and luteolin.

## 5 Conclusion

QCC can reduce blood content in feces and intestinal damage in coccidiosis infected chickens by regulating inflammation-related cytokines, reducing the intestinal inflammatory response, and preventing the development of parasites in intestinal epithelial cells, thereby possessing anti-coccidiosis activity.

## Data availability statement

The datasets presented in this study can be found in online repositories. The names of the repository/repositories and accession number(s) can be found in the article/supplementary material.

## Ethics statement

The animal studies were approved by the ethics approval reference number for the use of animals was XKY-20230420, which approved by the Chongqing Academy of Animal Science Animal Ethics Committee. The studies were conducted in accordance with the local legislation and institutional requirements. Written informed consent was obtained from the owners for the participation of their animals in this study.

## Author contributions

ZY: Writing – original draft. CC: Data curation, Writing – review & editing. SZ: Formal analysis, Investigation, Writing – original draft. HT: Resources, Writing – review & editing. MZ: Methodology, Writing – original draft. YY: Writing – original draft. HZ: Writing – original draft.

## Footnotes

^1^ https://www.uniprot.org/

^2^ https://www.genecards.org/

^3^ https://omim.org/

^4^ https://www.ncbi.nlm.nih.gov/

^5^ http://www.bioinformatics.com.cn/

^6^ https://string-db.org/

^7^ https://david.Ncifcrf.gov/

^8^ https://www.rcsb.org/
